# The neural basis of shared preference learning

**DOI:** 10.1093/scan/nsz076

**Published:** 2019-11-04

**Authors:** Harry Farmer, Uri Hertz, Antonia F de C Hamilton

**Affiliations:** 1 Institute of Cognitive Neuroscience, University College London, London, WC1N 3AZ, UK; 2 Department of Psychology, University of Bath, Bath, BA2 7AY, UK; 3 Department of Cognitive Sciences, University of Haifa, Haifa, 3498838, Israel

**Keywords:** fMRI, reinforcement learning, prediction error, self, social cognition

## Abstract

During our daily lives, we often learn about the similarity of the traits and preferences of others to our own and use that information during our social interactions. However, it is unclear how the brain represents similarity between the self and others. One possible mechanism is to track similarity to oneself regardless of the identity of the other (Similarity account); an alternative is to track each other person in terms of consistency of their choice similarity with respect to the choices they have made before (consistency account). Our study combined functional Magnetic Resonance Imaging (fMRI) and computational modelling of reinforcement learning (RL) to investigate the neural processes that underlie learning about preference similarity. Participants chose which of two pieces of artwork they preferred and saw the choices of one agent who usually shared their preference and another agent who usually did not. We modelled neural activation with RL models based on the similarity and consistency accounts. Our results showed that activity in brain areas linked to reward and social cognition followed the consistency account. Our findings suggest that impressions of other people can be calculated in a person-specific manner, which assumes that each individual behaves consistently with their past choices.

## Introduction

The ability to rapidly form and update our impressions about other people is a vital skill in navigating our complex social world. During our daily lives, we frequently learn about the traits and preferences of other people and use that information to inform our social interactions. However, the neural mechanisms that govern our learning of the relationship between our preferences and those of others are currently unclear. The current study investigated these mechanisms by combining fMRI and computational modelling.

Researchers investigating impression formation have sought to determine which brain areas respond when we learn about other people and when our expectations of others are violated. Most have done this by providing participants with some information about a novel person and then presenting either consistent information that confirms the previous impression or inconsistent one, which requires participants to update their impressions. These studies have shown increased activity in regions like the precuneus/posterior cingulate cortex (PCC), the temporal-parietal junction (TPJ) and the dorsomedial prefrontal cortex (dmPFC) when receiving inconsistent *vs* consistent information about another person’s moral behaviour (Mende-Siedlecki *et al.,* 2013a; Mende-Siedlecki and Todorov, 2016; Hughes *et al.,* 2017), competence (Ames and Fiske, 2013; Bhanji and Beer, 2013), traits (Ma *et al.,* 2012; Hackel *et al.,* 2015; Van der Cruyssen *et al.,* 2015) and political beliefs (Cloutier *et al.,* 2011). These regions are key nodes in the ‘mentalising’ network, which is activated when thinking about the beliefs, preferences and intentions of others (Adolphs, 2009; Van Overwalle, 2009; Frith and Frith, 2012; Schilbach, 2015).

The increased activation to inconsistent information seen in the mentalising network is reminiscent of the prediction error (PE) signal seen in reinforcement learning (RL) models. These signals compute the expectation of a future outcome (or reward) as being a function of the current expectation plus the product of the learning rate and the PE, i.e. the difference between the last expected and actual outcome (Behrens *et al.,* 2009; Ruff and Fehr, 2014). RL models have been shown to be biologically plausible both at the neurochemical level, where the pattern of midbrain dopamine neuron response matches that of reward PEs (Schultz, 2016), and at the level of whole brain anatomy (Botvinick *et al.,* 2011). This biological plausibility along with the findings outlined above have led researchers to suggest that regions in the mentalising network may be involved in calculating social PEs (Mende-Siedlecki *et al.,* 2013b; Hertz *et al.,* 2017; Wittmann *et al.,* 2018).

Several studies have investigated this possibility directly, using computational modelling to parametrically track PE from trial to trial and have found evidence of social PE tracking in the dmPFC, the anterior cingulate cortex (ACC), the TJP, the superior temporal sulcus (STS), the medial temporal gyrus (MTG), ventrolateral PFC (vlPFC) and the precuneus (Behrens *et al.,* 2008; Hackel *et al.,* 2015; Stanley, 2016; Lockwood *et al.,* 2018). A recent study by Wittmann *et al.* (2016) examined the related phenomenon of self-other mergence, in which knowledge about another person’s performance reciprocally influences judgements of one’s own performance. They found a division between PEs for self-performance, represented in the anterior cingulate cortex, and PEs for other performance, represented in the dmPFC. Interestingly, individual variance in the strength of dmPFC activation also predicted how far participants’ self PEs were affected by the performance of the others. Such findings have led some researchers (e.g. Bach and Schenke, 2017; Joiner *et al.,* 2017) to argue that predictive processing plays a key role in social cognition.

To date, most studies examining social PEs have considered cases where participants learn about other individuals, but do not examine the relationship between those individuals and the self (although see Will *et al.,* 2017 for an interesting exception). A distinct literature has examined the role of self-similarity in impression formation (Boer *et al.,* 2011; Montoya and Horton, 2013) and shown that self-similarity can lead to liking and affiliation. Numerous studies have shown that those we perceive as similar to us in terms of traits (Paunonen and Hong, 2013), attitudes (Montoya and Horton, 2013) and preferences (Boer *et al.,* 2011) tend to be evaluated more favourably than those perceived as different. There is evidence for a ventral–dorsal gradient in the mPFC when processing the similarity of others with similar others being processed in the ventromedial prefrontal cortex (vmPFC) and dissimilar others in the dmPFC (Denny *et al.,* 2012; Sul *et al.,* 2015).

The current study aims to test how the brain tracks and learns about other people from the self-similarity of their choices. In particular, we distinguish two possible ways in which the brain could track others: the similarity approach and the consistency approach. The similarity approach assumes that, on each trial, we consider ‘is this person like me on this trial?’ and assign high PEs to any trial where an agent makes a different choice to me. The consistency approach assumes that we model each person we encounter as an individual with a level of overall similarity to me. On each trial, we then consider ‘is this person’s choice consistent with their overall similarity to me?’ and assign high PEs to any trial where the agent behaves in a way that is inconsistent with that agent’s track record.

To do this, we adapted RL models to investigate how the brain tracks the choices of two different agents in terms of how similar they are to the participant’s own choices. It is important to note that we are not claiming that the tracking of similarity is necessarily linked to reward-based reinforcement in a direct manner. Rather, we use RL models because they can track the accumulation of information and evidence over time. This allows us to look at how the brain represents confirming and disconfirming information about other’s similarity to ourselves. For a related approach applied to the learning of others’ traits, see Zaki *et al.* (2016).

**Fig. 1:**
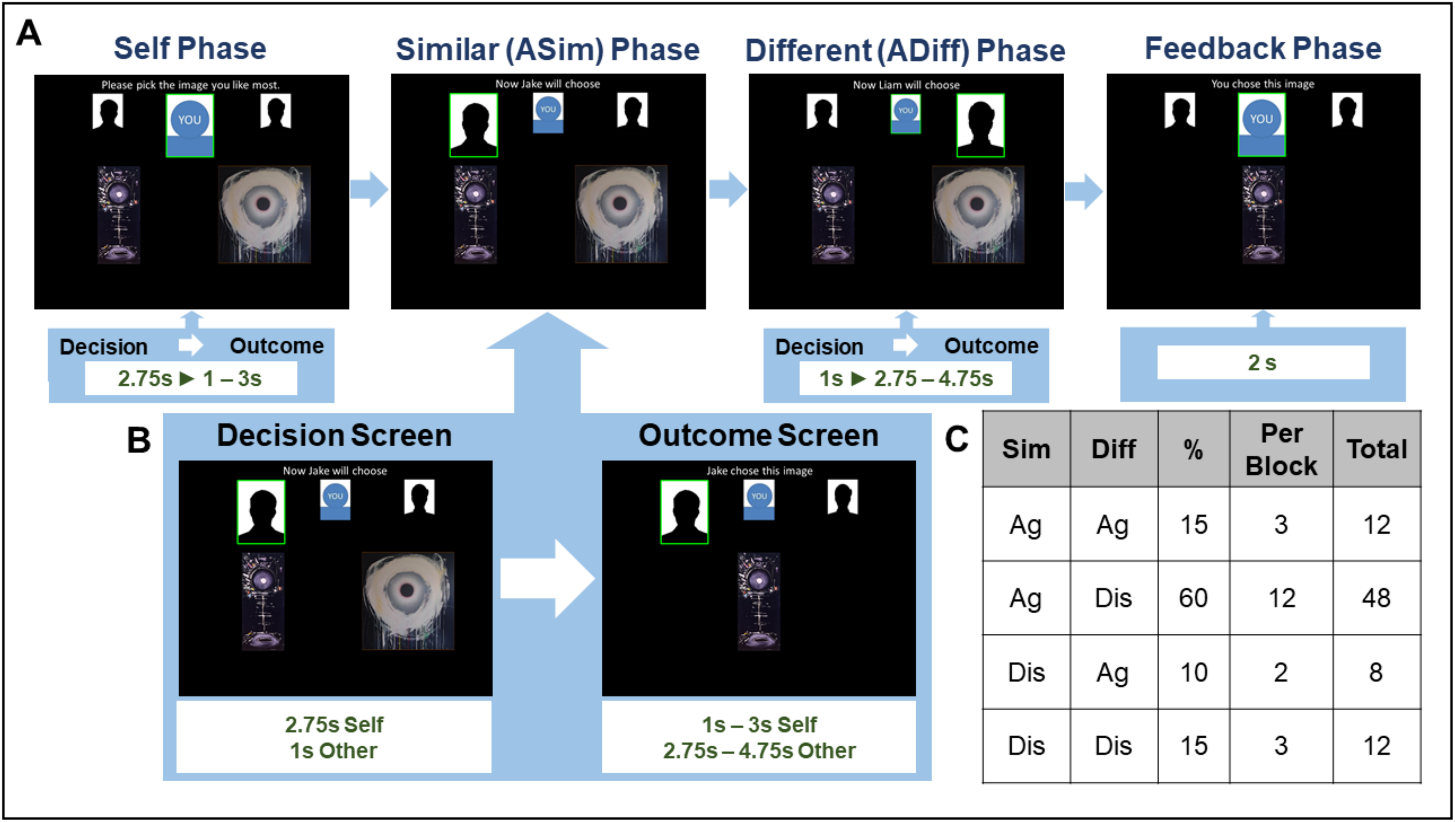
Outline of experimental trial structure and number of trials per condition. A trial phases and timings. Each trial has four phases (self, similar, different, feedback). On every screen, three icons at the top represent the participant (blue outline in the centre) and the two agents (two photos), with one icon enlarged in a green square to show who is the ‘active player’ in this phase. In the self-phase, participants chose which of two pictures they prefer. In the ASim phase and ADiff phase, the two agents ASim and ADiff chose pictures and the participant sees the outcome. The order of these two phases was counterbalanced. Finally, in the Feedback phase, the participant sees a reminder of his/her own choice. **B. Detail of one phase**. This shows an expanded view of the two different screens within the ASim phase; the same structure was used for the Self phase and ADiff phase. Participant’s first see a ‘decision screen’ with the two pictures used on this trial. During the decision screen participants either chose their own preferred painting (Self phase) or waited to see the choice of the agent (similar and different phases). Then they see an ‘outcome screen’ which shows either the painting they chose (Self phase) or the painting the agent chose (ASim and ADiff phases). The durations of each screen are given at the bottom of the figure, and multiple times separated by a dash represent the jittering in order to effective temporal sampling resolution much finer than one TR**. C. Number of trials of each type.** This table shows the breakdown of the four possible combinations of choices made by the two agents, ASim and ADiff. Each agent could agree with the participant’s choice (Ag) or disagree (Dis). The columns show the percentage of trials, number of trials by block and total number of trials which had a particular pattern of choices.

Our task created a context in which participants chose which painting they prefer (an arbitrary aesthetic choice) and then learn the preferences of two agents for the same paintings (see Figure Fig. 1). Using fMRI and computational modelling, we can identify which brain areas track agents’ preferences relative to self-preferences in a trial-by-trial manner. In each trial, our participants saw two paintings and indicated which they preferred. They then saw the preferences of two agents, a similar agent (ASim) who chose the same painting 75% of the time and a different agent (ADiff) who chose the same painting 25% of the time. Using RL models, we are able to calculate the prior probability of the agents’ choice and the PE of their actual choice separately for each trial and each agent, allowing us to localise brain regions where BOLD signal tracks the model parameters.

**Fig. 2:**
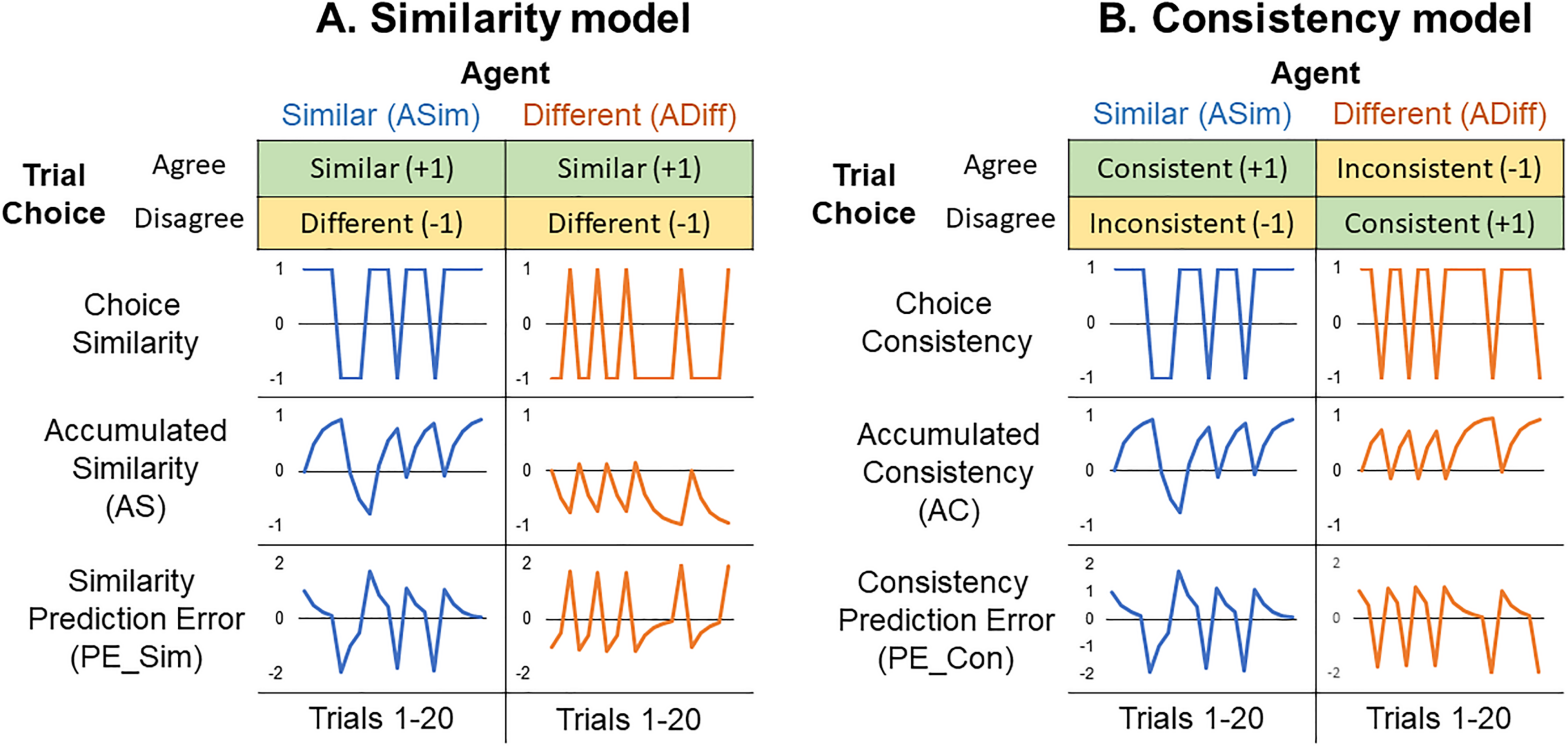
Two possible ways that the choices of the two agents, ASim and ADiff, may be tracked in the brain**. A. Similarity approach.** The yellow/green boxes in the top row show how trials are classified as Similar or Different according to whether the agent choose the same picture as the participant or not, and the same classification is used for both agents. Green indicates that a choice is given a positive value and yellow that it has a negative value. This is reflected in the sample sequence of 20 trials, where the ‘choice similarity’ tends to be high for ASim and low for ADiff. Based on the choice similarity, the Sim_PE and AS parameters are calculated as in equations 1 and 2. **B. Consistency approach.** Trials are classified as Consistent or Inconsistent according to whether the agent conforms to type. Both agents show high choice consistency most of the time in the sample of 20 trials shown below. Based on the choice consistency the PE_Con and AC parameters are calculated as in equations 3 and 4.

We then used RL to create signed PE models of both the similarity and consistency approaches to tracking the agent’s choices (see Figure Fig. 2). In the similarity model, agents are tracked only in relation to the participant’s own preferences, on a single dimension of ‘distance from me’. This means that the model will tend to have positive PEs for ASim and negative PEs for ADiff (see Figure Fig. 2A). In the RL model, each signed PE then contributes to an accumulated similarity (AS) parameter, which will tend to be high for ASim (who is often similar) and low for ADiff (who is often different). To make this model clear, we term the two parameters the ‘similarity PE’ (PE_Sim) and the AS.

The alternative is the consistency model, which assumes that participants track agents and choices in terms of whether the agent’s choice is consistent with their past level of preference similarity to the participant. Thus, we label each agent’s choices as ‘consistent’ or ‘inconsistent’ with that agent’s past behaviour: agreeing with the participant is *consistent* for ASim but *inconsistent* for ADiff. In this model, a trial will have negative consistency PE when ASim chooses a different picture to the participant, because this is unlike ASim’s typical preference. In the same way a trial will have negative PE when ADiff chooses the same picture as the participant (unlike ADiff’s typical preference) (see Figure Fig. 2B). These PEs feed into the accumulated consistency (AC) of each agent, which will be high when that agent conforms to type (i.e. high for both ASim and ADiff most of the time) but will fall if the agent makes atypical choices. To make this model clear, we term the two parameters the ‘consistency PE’ (PE_Con) and the AC.

Importantly, these two models predict a different pattern of brain activity in our experimental design, as ASim and ADiff’s trial-by-trial preferences can have the same sign (both consistent, according to the consistency approach) or opposite sign (as they chose different images, according to the similarity approach, see Figure Fig. 2). It is important to note that while our study can test how well each of these models fit activation in different brain areas, we are not claiming that they are mutually exclusive competing accounts. Indeed, it is entirely plausible that some brain areas track similarity of choices directly while others track the consistency of choices. Our design allows for us to investigate the neural signature of both models, in two separate GLMs, and thus identify which brain areas (if any) are involved in each of these two ways of processing similarity relationships.

## Methods

### Design

In our study, participants tracked the choices of two agents on multiple trials, in relation to their own choices. On each trial, the participant and two agents, ASim and ADiff, indicated which of two paintings they preferred. ASim chose the same painting as the participant in 75% of all trials, while ADiff only chose the same painting in 25% of trials.

### Participants

Twenty-five participants (mean age ± SD: 25.1 ± 5.7, 11 male) took part in this study, which was approved by the University College London, Institute of Cognitive Neuroscience Research Department’s Ethics Committee. All participants gave their informed consent to participate and were paid for their participation. All participants were right handed and were screened for neurological disorders. Due to technical issues, pre- and post-ratings data were lost for seven participants. Therefore, our final sample size for the ratings analysis was *n* = 18. As we did not use this ratings data for model fitting, and data on all 25 participant’s choices during the task were collected, this issue did not impact on the fMRI analysis so the full sample *n* = 25 was used for fMRI analysis.

## Procedure

### Experimental task

The main task in this study was an aesthetic choice task. Participants were told that in each trial, they would see a pair of paintings (see Supplementary Materials S1.1) and would have to choose which painting they preferred. They were informed that other participants had previously indicated which of the paintings they preferred and that they would see the choices of two previous participants during the study. Names and faces were assigned to these ‘previous participants’, but in fact they were computer agents whose choices were determined based on the participant’s own choices. Prior to entering the scanner, participants completed a training block of the task (see Supplementary Materials S1.2). After the training, participants learnt the names of the agents with whom they would do the experimental task. They also rated their faces for similarity, likeability and attractiveness, using a 10-point scale in order to provide us with a manipulation check as to how well the participants learnt the similarity of the agent to themselves. Other than being asked to rate their similarity to the agent, participants were not given any information to suggest the relationship between their choices and those of the agents were important to the task.

Each trial was divided into four phases (see Figure Fig. 1A). The first three phases were each split into two screens, a *decision screen* and an *outcome screen* (see Figure Fig. 1B). In the self-phase, participants were shown a pair of paintings on the *decision* screen and had 2.75 s to choose which they preferred using the left and right buttons on a response box. They then saw an *outcome* screen displaying their preferred painting for a jittered interval (1–3 s). In the similar phase, participants first saw a 1-s *decision* screen, which displayed the pair of paintings along with an indicator that ASim was choosing. This was followed by an *outcome* screen, which displayed the agent’s preferred painting for a jittered interval (2.75–4.75 s). In the different phase, participants again saw a *decision* screen with an indicator that ADiff was choosing, followed by a jittered *outcome* screen displaying that agent’s preferred painting. The order of the similar and different phases was pseudorandomised across trials. Finally, each trial contained a *feedback* phase in which participants again saw their own choice for an interval of 2 s.

Participants completed four sessions of 20 trials (see Figure Fig. 1C for a breakdown of trial types by block); at the end of each block, they rated the similarity, likeability and attractiveness of each agent using a 10-point scale. Using fast event-related design, i.e. varying the intervals of the outcome screen in the three choice phases and using many trials, an effective temporal sampling resolution much finer than one TR for each of these periods was achieved. The lengths of the intervals were uniformly distributed for each period, ensuring that evoked haemodynamic responses time locked to the events were sampled evenly across the time period following each choice period.

### Model-based fMRI analysis

For full details of image acquisition and fMRI data analysis, please see Supplementary Materials S1.3. To examine whether the relationship between the participant preferences and those of the agents was coded in terms of similarity or consistency, two general linear models (GLM) were created, which include different trial types and the parameters of the two RL models. Both GLMs modelled BOLD activation during *outcome* screen for ASim and ADiff separately. Regressors of no interest modelled activity during the *self-choice outcome* screen, the *feedback* phase, the ratings periods and trials where participants failed to make a choice and the residual effects of head motion. In addition, parametric modulators linked to the *outcome* screen regressors allowed us to model the values of our RL parameters on a trial-by-trial basis. Note that we also conducted a more traditional GLM without RL parameters, the details of which can be found in Supplementary Materials S2.

In the similarity GLM, we modelled the signed similarity PE (PE_Sim) and accumulated similarity (AS) between the agent choice and the participant choice for each agent (*n*), using the following algorithms:

[1] \begin{equation*}\mathrm{PE}\_{\mathrm{Sim}}_n(t)=\mathrm{ChoiceSim}(t)-{\mathrm{AS}}_n(t)\end{equation*}

[2] \begin{equation*}{\mathrm{AS}}_n\left(t+1\right)={\mathrm{AS}}_n(t)+\lambda \ast \mathrm{PE}\_\mathrm{Sim}(t)\end{equation*}

where

$$\mathrm{ChoiceSim}(t)=\left\{\begin{array}{c}\kern-2.5em 1\kern0.5em \mathrm{agent}\kern0.17em \mathrm{chose}\kern0.17em \mathrm{same}\kern0.17em \mathrm{picture}\;\mathrm{as}\;\mathrm{participant}\\{}\kern-.2em\hbox{-} 1\kern.2em \mathrm{agent}\kern0.17em \mathrm{chose}\kern0.17em \mathrm{different}\kern0.17em \mathrm{picture}\kern0.17em \mathrm{from}\kern0.17em \mathrm{participant}\end{array}\right.$$

As we did not fit the model to any response, we set the learning rate (λ) with a fixed value of 0.5 and initial AS was set to 0. The learning rate of 0.5 was chosen a priori and fixed for all participants, to indicate the carry-on effect of previous trials to the current trials. This value was chosen because it is in the middle of the LR range (0–1) and indicates a decaying memory window of about four trials. We chose this conservative approach and did not explore learning rates further to avoid double dipping the data or *post hoc* analysis. AS was set at 0 as this represented no a priori expectation of a similarity relationship between the participant and the agents. In total, there were six regressors-of-interest in our similarity GLM: outcome screens, AS values, and PE_Sim values for both ASim and ADiff.

In the consistency GLM, we modelled the signed consistency PE (PE_Con) and AC between the agent choice and the participant choice for the two agents (*n* = ASim or ADiff), using the following algorithm.

[3] \begin{equation*}\mathrm{PE}\_{\mathrm{Con}}_n(t)=\mathrm{ChoiceCon}(t)-{\mathrm{AC}}_n(t)\end{equation*}

[4] \begin{equation*}{\mathrm{AC}}_n\left(t+1\right)={\mathrm{AC}}_n(t)+\lambda \ast \mathrm{PE}\_{\mathrm{Con}}_n(t)\end{equation*}

where

$$\mathrm{ChoiceCont}(t){=}\left\{\begin{array}{c}\kern-1.5em1\kern1.5em \mathrm{Agent}\hbox{'}\mathrm{choice}\kern0.17em \mathrm{was}\kern0.17em \mathrm{consiststent}\kern0.17em \mathrm{with}\kern0.17em \mathrm{the}\mathrm{ir}\\\kern0.17em \mathrm{overall}\; {}\mathrm{s}\mathrm{imilarity}\kern0.17em \mathrm{to}\kern0.17em \mathrm{the}\kern0.17em \mathrm{participant}\hbox{'}\mathrm{s}\;\mathrm{choice}\\{}\hbox{-} 1\kern1em \mathrm{Agent}\hbox{'}\mathrm{s}\;\mathrm{choice}\kern0.17em \mathrm{was}\kern0.34em \mathrm{inconsitent}\kern0.17em \mathrm{with}\kern0.17em \mathrm{the}\mathrm{ir}\\{}\mathrm{overall}\kern0.17em \mathrm{similairty}\kern0.17em \mathrm{to}\kern0.17em \mathrm{the}\kern0.17em \mathrm{participant}\hbox{'}\mathrm{s}\;\mathrm{choice}\mathrm{s}\end{array}\right.$$

Again, the learning rate (λ) was set to 0.5 and initial AC was set to 0 (see Figure Fig. 2C for examples of how AS and PE varied across 20 trials). In total, there were six regressors of interest in our consistency GLM: outcome screens; AC values and PE_Con values for both ASim and ADiff.

## Results

### Behavioural results

**Fig. 3:**
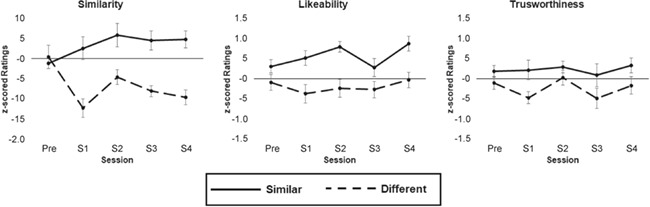
Z-scored ratings of liking similarity and trustworthiness for the similar and different agents across rating sessions.

To examine whether learning about the preferences of the agents changed participants’ feelings of affiliation towards them, we collected ratings of similarity, likeability and trustworthiness at the start of the study and after every 20 trials. This meant that each participant contributed five ratings of each of the three attributes across the study. These ratings were then z-scored within participant to remove baseline differences between participants, before the next analysis. Three separate 2 (agent: similar/different) × 5 (session number: pre/S1/S2/S3/S4) repeated measures ANOVAs were carried out on the z-scored ratings of similarity, liking and trust (see Figure Fig. 3). Due to problems with data recording, the ratings from seven participants were incomplete and were excluded from the behavioural analysis leaving a remaining sample of 18 participants.

The ANOVA on similarity ratings found a significant main effect of agent, *F*(1.17) = 23.52, *P* < 0.001, η^2^_p_ = 0.58. Overall participants rated ASim as being more similar (*M* = 0.33, *MSE* = 0.15) to them than ADiff (*M* = −0.68, *MSE* = 0.12). There was also a significant interaction between agent and session *F*(1.17) = 5.65, *P* = 0.001, η^2^_p_ = 0.25. To examine this interaction further, ratings for ADiff were subtracted from the ratings of ASim for each session to create a difference score. Pairwise comparisons (Bonferroni corrected) showed that the difference score for the pre-session (*M* = −0.16, *MSE* = 0.36) significantly differed from the scores after sessions S1 (*M* = 1.49, *MSE* = 0.35), *P* < 0.05, S3 (*M* = 1.26, *MSE* = 0.29), *P* < 0.05, and S4 (*M* = 1.43, *MSE* = 0.25), *P* < 0.01. No other pairwise comparisons were significant.

The ANOVA on liking ratings found a significant main effect of agent, *F*(1.17) = 23.8, *P* < 0.001, η^2^_p_ = 0.58. Overall participants rated ASim as being more likeable (*M* = 0.55, *MSE* = 0.07) than ADiff (*M* = −0.2, *MSE* = 0.12). There was no significant effect of session and no interaction between session and agent. The ANOVA on trust ratings found a significant main effect of agent, *F*(1.17) = 7.67, *P* < 0.05, η^2^_p_ = 0.31. Overall participants rated ASim as being more trustworthy (*M* = 0.23, *MSE* = 0.11 than ADiff (*M* = −0.24, *MSE* = 0.01). There was no significant main effect of session and no interaction between session and agent.

### fMRI results

#### Main effect of agent preference similarity

**Fig. 4:**
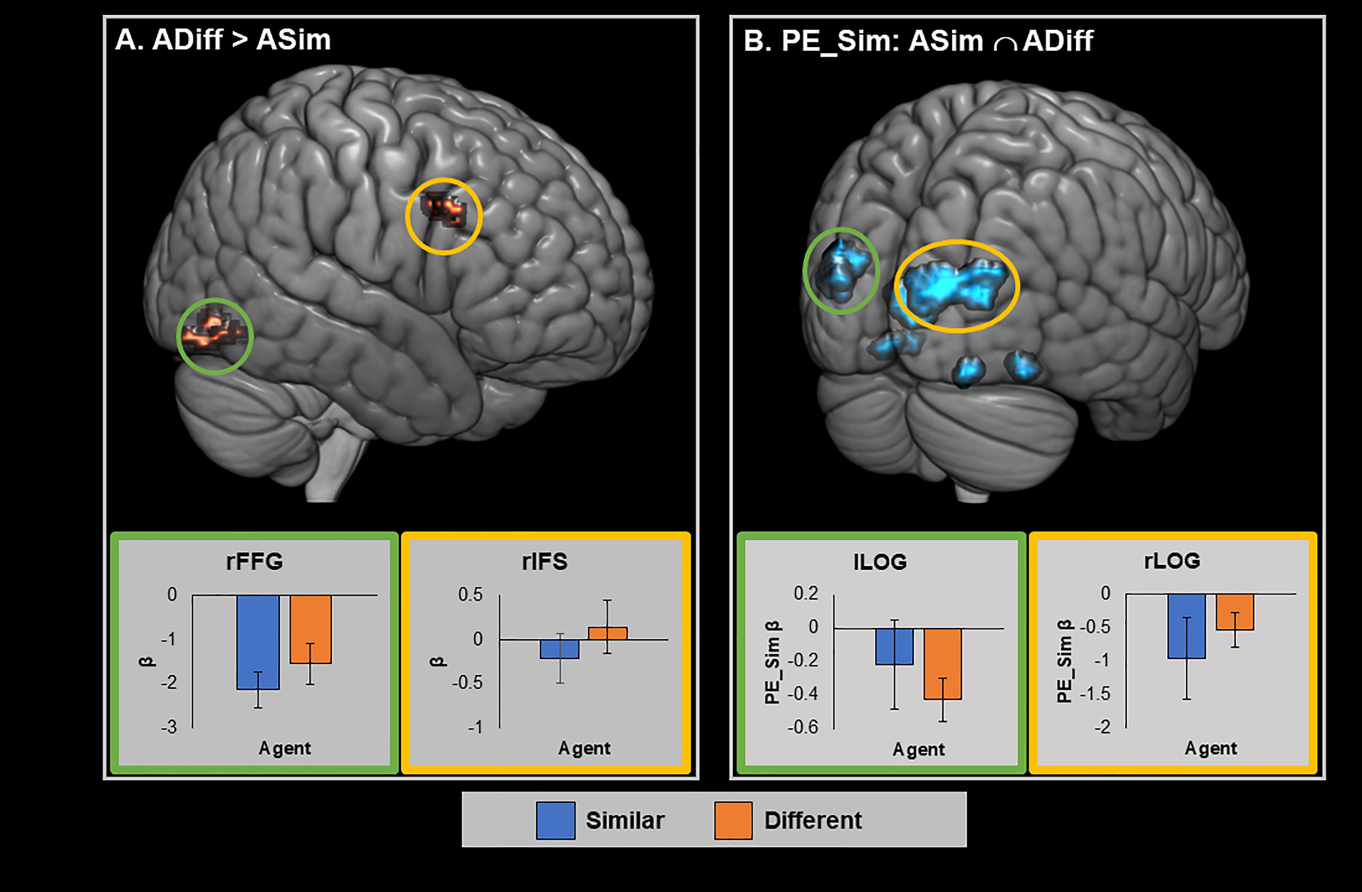
A. Brain areas showing significant cluster corrected results in the ADiff > ASim contrast for the Outcome screen. B. Brain areas tracking the PE_Sim parameter (similarity PE) for the outcome screen across both agents, cluster corrected. Parameter estimates in the lower panel are averaged across the whole cluster. Error bars represent SEM. Graph border colours indicate matching circled area. Red/yellow represents positive activations and blue/green represents negative activations.

Two contrasts investigated the main effect of agent identity (ASim/ADiff) on BOLD response. The regressors, which contribute to these contrasts, were identical in the similarity GLM and the consistency GLM, so the results here are the same for both. The ADiff > ASim contrast revealed that observing the choice of ADiff compared to ASim led to a greater activation in the right inferior frontal sulcus (rIFS) and in a cluster centred on the right fusiform gyrus (rFG) (Table 1 and Figure Fig. 4A). No significant activations were found in the ASim > ADiff contrast.

**Table 1:** Peak voxel coordinates in MNI space, *z*-values and cluster sizes for analyses of the outcome screen showing significant effects after cluster correction for main effect of similarity. Same shading indicates local maxima in distinct anatomical regions within the same cluster, BA indicates Brodmann area and *k* indicates the cluster size threshold for whole brain significance of *P* < 0.05

Region	Hem.	*X*	*Y*	*Z*	*Z*-Score	Cluster size
Different > similar (*k* = 33)						
Inferior frontal sulcus (BA 44)	R	38	10	34	3.86	57
Fusiform gyrus (BA 18)	R	14	−82	−10	3.51	72
Lateral occipital gyrus (BA 19)	R	30	−82	−14	3.40	

#### Parametric analysis of the similarity GLM

To identify brain regions, which tracked accumulated similarity (AS) across both agents, we calculated a conjunction of the RL parameters for each of the agents, that is AS_ASim_ ∩ AS_ADiff_. This did not reveal any significant clusters in either a positive or negative direction, suggesting that no brain areas directly tracked preference similarity between agents and participant. Similarly, there were no significant clusters that tracked the positive conjunction of similarity PE for both agents, that is, PE_Sim_ASim_ ∩ PE_Sim_ADiff_. This means that no areas showed increased activation when both agents preferences were unexpectedly similar to that of the participant. However, the negative PE_Sim conjunction analysis revealed that unexpected dissimilarity between either agent choice and participant choice correlated with activation in a number of clusters within the occipital cortex including the bilateral lateral occipital cortex (LOC) and the lingual gurus (Table 2 and Figure Fig. 4B).

**Table 2:** Peak voxel coordinates in MNI space, *z*-values and cluster sizes for analyses of the outcome screen in the similarity GLM showing significant effects after cluster correction for conjunction analyses of the AS and PE parametric modulators. Same shading indicates local maxima in distinct anatomical regions within the same cluster, BA indicates Brodmann area and *k* indicates the cluster size threshold for whole brain significance of *P* < 0.05

Region	Hem.	*X*	*Y*	*Z*	*Z*-Score	Cluster size
Negative PE_Sim similar ∩ different (*k* = 42)						
Lateral occipital gyrus (18)	L	−28	−94	16	4.06	324
Lateral occipital gyrus (37)	R	32	−54	−16	3.81	86
Lateral occipital gyrus (18)	R	24	−90	18	3.80	457
Middle occipital gyrus (19)	R	36	−80	22	3.74	
Lingual gyrus (17)	L	−6	−78	8	3.79	249
Lateral occipital gyrus (19)	R	28	−82	−16	3.67	64
Lateral occipital gyrus (37)	L	−28	−60	−16	3.54	100
Fusiform gyrus (37)	L	−26	−48	−14	3.39	

### Parametric analysis of the consistency GLM

**Fig. 5:**
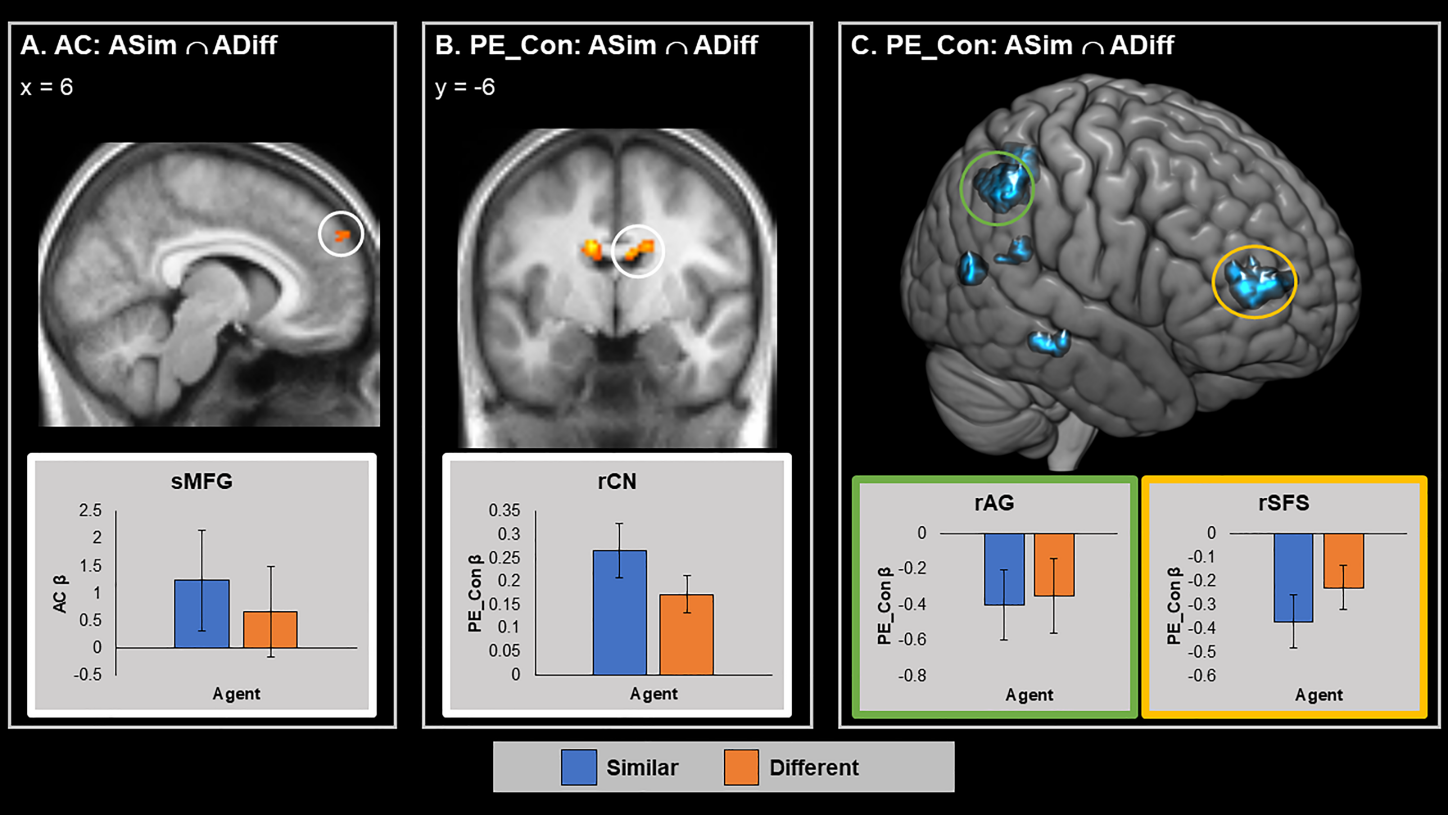
Brain areas showing significant cluster corrected tracking of AC and PE_Con for the Outcome screen. A. Areas significantly tracking AC in the positive ASim ∩ ADiff conjunction. B. Areas significantly tracking PE_Con in the positive ASim ∩ ADiff conjunction. C. Areas significantly tracking PE_Con in the negative ASim ∩ ADiff conjunction. Parameter estimates averaged across whole cluster. Error bars represent SEM. Graph border colours indicate matching circled area. Red/yellow represents positive activations and blue/green represents negative activations. sMFG = superior medial frontal gyrus, rCN = right caudate nucleus, rAG = right AG, rSFS = right superior frontal sulcus.

To identify brain regions tracking the consistency of agents’ choices across both agents, we first examined the conjunction of areas tracking AC, that is AC_ASim_ ∩ AC_ADiff_. The positive conjunction showed a significant activation in a cluster-corrected region centred on the superior medial frontal gyrus (smFG) (Table 3 and Figure Fig. 5A). This region showed greater activation as evidence for the consistency of the agents’ choice similarity to the self-increased, and lower activation during inconsistence periods. No significant activations were found in the conjunction analysis testing for areas negatively correlated with AC.

**Table 3:** Peak voxel coordinates in MNI space, *z*-values and cluster sizes for analyses of the outcome screen in the consistency GLM showing significant effects after cluster correction for conjunction analyses of the AS and PE parametric modulators. Same shading indicates local maxima in distinct anatomical regions within the same cluster, BA indicates Brodmann area and *k* indicates the cluster size threshold for whole brain significance of *P* < 0.05

Region	Hem.	*X*	*Y*	*Z*	*Z*-Score	Cluster size
Positive AC ASim ∩ ADiff (*k* = 43)						
Superior medial frontal gyrus (9)	R	8	56	34	3.37	76
Superior medial frontal gyrus (10)	L	−2	54	24	3.25	
Superior medial frontal gyrus (10)	R	6	56	22	3.17	
**Positive PE_Con ASim ∩ ADiff (*k* = 42)**						
Corpus callosum	L	−12	−6	28	4.54	52
Caudate nucleus	R	16	−6	28	3.90	71
Corpus callosum	L	−4	14	12	3.64	56
**Negative PE_Con ASim ∩ ADiff (*k* = 42)**						
AG (40)	R	56	−46	50	4.22	341
Interparietal sulcus (40)	R	32	−50	40	3.70	
Superior frontal sulcus (10)	R	34	50	10	4.20	270
Superior temporal sulcus (37)	R	60	−58	16	3.85	76
Superior temporal sulcus (41)	R	44	−42	20	3.72	43
Superior temporal sulcus (39)	R	42	−54	16	3.19	
Precuneus (39)	R	10	−56	48	3.72	106
Middle temporal gyrus (21)	R	60	−20	−16	3.46	57
Superior temporal sulcus (21)	R	62	−28	−10	3.43	

The conjunction analysis testing for areas tracking PE in consistency (PE_Con_ASim_ ∩ PE_Con_ADiff_) identified significant cluster-corrected activations bilaterally in a dorsal region of the caudate nucleus as well as in a more ventral midbrain region of the left hemisphere (Table 3 and Figure Fig. 5B). These areas showed increased BOLD response when the agents’ choices were unexpectedly consistent with their overall preference, and decreased activation when agents’ choices were unexpectedly inconsistent. Note that while the peak activation in the more dorsal left hemisphere cluster is in fact found in the neighbouring corpus callosum, both dorsal clusters showed considerable overlap with the caudate nucleus. The conjunction analysis testing for areas tracking PE_Con in a negative direction identified significant clusters in several right hemisphere regions, namely the angular gyrus (rAG), the superior frontal sulcus (rSFS), the rSTS, the rMTG and the precuneus (Table 3 and Figure Fig. 5C). These areas showed increased BOLD response when the agents’ choices were unexpectedly inconsistent with their overall preference, and reduced activity when the agents’ choices were highly predictable.

## Discussion

Our study examined the neural basis of learning about preference similarity between self and others and its role in promoting affiliation. We created a context where participants could express a preference for a painting and learn about the preferences of two agents for the same paintings. Our behavioural data show that similar preferences lead to higher ratings of liking, trustworthiness and similarity, indicating that participants tracked the agents’ preferences in relation to their own preferences.

Our introduction outlined two possible, non-mutually exclusive, ways in which preference similarity might be tracked in the brain: either by a general mechanism, which tracks an agent’s choice in relation to one’s own, i.e. how similar or dissimilar they are from the self, or via a model of consistency, which tracks agent’s choices in terms of their consistency to that agent’s previous choice, i.e. how *consistently* similar or dissimilar they are from the self. To examine the evidence for each of these two mechanisms, we created two RL models, which tracked the agents’ choices based on similarity and consistency, respectively. Our results from the similarity model indicated that regions of the visual cortex negatively tracked similarity PE (PE_Sim). Results from the consistency model showed a number of brain areas tracking different variables associated with the consistency model; the dorsomedial pre-frontal cortex (dmPFC) tracking AC, and the caudate nucleus, AG and precuneus tracked consistency PE (PE_Con). The caudate is involved in value updating (O’Doherty *et al.,* 2004; Bhanji and Delgado, 2014), while the AG and precuneus are associated with social cognition (Spreng *et al.,* 2009; Murray *et al.,* 2015). Below, we elaborate on the results of the AC conjunction before moving on to discuss the findings on PE_Con and PE_Sim.

### dmPFC tracks AC

The AC parameter represents a trial-by-trial estimate of the probability that a person makes choices in line with his previous choices, this is, that the similar agent (ASim) should choose the same painting as the participant while the different agent (ADiff) should choose differently. The only area we found tracking AC was a cluster in the bilateral superior medial frontal gyrus (smFG) corresponding to the anterior region of the dmPFC. The dmPFC is known to be a key area for the processing of information about both self and other (Amodio and Frith, 2006; Eickhoff, Laird, Fox, Bzdok, and Hensel, 2016; Mitchell, Banaji, and Macrae, 2005). See Supplementary Materials S3 for a more detailed survey of previous results.

The dmPFC’s involvement in coding prior knowledge of other people is supported by previous research suggesting that the dmPFC encodes reputational priors of one’s partners during economic games (Hampton *et al.,* 2008; Fouragnan *et al.,* 2013). Our results build on these findings by suggesting that dmPFC PEs track the *consistency* of the agent’s similarity to the self rather than simply tracking preference similarity.

### Consistency PEs are tracked by regions involved in reward and social cognition

PE_Con reflects the difference between the agent’s choice and the participant’s expectation of what choice the agent will make. For example, the model assigns a positive update signal when ADiff picked the painting not chosen by the participant, and a negative signal when ADiff picked the same painting (see Figure Fig. 2). Areas that tracked PE_Con revealed two distinct patterns of activation. Clusters in the bilateral caudate nucleus (Fig. 4B) showed increased activity when the agents chose consistently with their type. Meanwhile, clusters in regions associated with social cognition including the superior temporal sulcus (STS), the AG, precuneus and superior frontal sulcus (SFS; Figure Fig. 4C) showed increased activations when the agent’s choice was inconsistent with their type. Overall, this pattern shows that PE tracking in these regions is not a ‘generic’ signal of how similar a person is to me, but rather reflects how much each person’s choice conforms to their typical pattern of similarity to me.

The caudate nucleus, along with other parts of the striatum, has been heavily implicated in the generation of PEs during RL of rewards for self (O’Doherty *et al.,* 2004; Balleine *et al.,* 2007; Schultz, 2015) and others (Báez-Mendoza and Schultz, 2013; Bhanji and Delgado, 2014; Ruff and Fehr, 2014). Previous studies have shown that the caudate nucleus is also involved in signalling PEs when learning the characteristics of others. King-Casas *et al.* (2005) found that the caudate nucleus activity tracked PEs regarding the trustworthiness of other during an economic game. Subsequent studies have found similar results for trustworthiness (Fareri *et al.,* 2012; Fouragnan *et al.,* 2013; Fett *et al.,* 2014), generosity (Fareri *et al.,* 2012), reliability in advice giving (Diaconescu *et al.,* 2017) and general behavioural traits (Mende-Siedlecki and Todorov, 2016). Our findings add to this literature by showing that caudate nucleus activity also tracks PE when learning about the similarity of others’ preferences to one’s own.

The regions showing greater activations when PE_Con was negative, i.e. when the agents’ choice was inconsistent with their typical choices, are key nodes of the mentalising network involved in processing information about self and others (Spreng *et al.,* 2009; Van Overwalle, 2009; Barrett and Satpute, 2013; Murray *et al.,* 2015). These areas have been implicated in the formation of impressions about other peoples’ traits (Gilron and Gutchess, 2012; Hackel *et al.,* 2015; Hughes *et al.,* 2017; Ma *et al.,* 2012; Mende-Siedlecki *et al*., 2013b), beliefs (Cloutier *et al.,* 2011) and abilities (Bhanji and Beer, 2013; Mende-Siedlecki *et al*., 2013a). Of particular note are two studies which directly modelled PEs for learning about the traits of other. Hackel *et al.* (2015) found that the precuneus and STS tracked PEs for other generosity during an economic game, while Stanley (2016) found that only the precuneus showed greater tracking of PEs in a social verses non-social setting. The current study shows that these regions also track PEs regarding the similarity relationship between self and others, underlining the role of PEs in social learning (Joiner *et al.,* 2017).

It is also notable that while previous studies on social impression formation have tended to show bilateral activations of the mentalising network, in the current studies, activity was limited to the right hemisphere. This is consistent with previous research demonstrating right lateralisation for tasks involving self and other differentiation (Decety, 2003; Uddin *et al.,* 2005; Kaplan *et al.,* 2008; Hu *et al.,* 2016).

### Similarity-related responses in regions involved in visual attention

In addition to modelling the RL parameters, we also directly contrasted the outcome screen where participants see the choices of ASim with the outcome screen for ADiff. This contrast shows greater activation for ADiff in two clusters: one centred on the rIFS and the other on the rFG. The IFS has been implicated in attentional processing and in particular in the control of attentional shifts by both internal goals and by salient external stimuli (Aron *et al.,* 2004, 2014; Asplund *et al.,* 2010; Levy and Wagner, 2012; Filimon *et al.,* 2013), while the FG is known to play a key role in the visual perception of faces (Rotshtein *et al.,* 2005; Kanwisher and Yovel, 2006; Contreras *et al.,* 2013). Interestingly, a previous study found greater FG activation when participant observed faces of individuals judged to have different traits to themselves (Leshikar *et al.,* 2016). These findings were also consistent with our conjunction analysis of regions that showed a negative relationship to the value of PE_Sim. This analysis revealed that when an agent made an unexpectedly dissimilar choice to that of the participant, it led to increased activation across a series of visual areas including regions in the bilateral LOC and in the left FG.

The activation of these areas suggests that participants may have found the choices of ADiff to be more attention-grabbing than those of ASim in a comparable way to studies that have demonstrated an attentional bias towards untrustworthy as opposed to trustworthy agents (Vanneste *et al.,* 2007; Dzhelyova *et al.,* 2012; Farmer *et al.,* 2016).

### Comparison with non-RL GLM

In addition to running our main RL analysis, we also conducted a more traditional GLM, which divided our trails using a 2 × 2 design with confederate/agent identity (similar *vs* different) as one factor and choice decision (agree *vs* disagree) as the other factor, the interaction between them (i.e. similar agree and different disagree *vs* similar disagree and different agree) was equivalent to our consistency model. This allowed us to compare the results of our RL model to more traditional non-parametric approaches (see Supplementary Materials S2 for full details and results). When comparing the results of the RL models and the conventional GLM the activations for the choice main effects and the consistency (interaction effects) were largely similar with the disagree > agree contrast showing activations equivalent to the clusters shown for areas that negatively tracked similarity PEs, the consistent > inconsistent contrast showing activations for two of the three clusters we identified that positively tracked consistency PE and the results for the inconsistent > consistent contrast showing results largely consistent with areas negatively tracking consistency PE.

Despite these similarities, our model has two advantages over the non-RL GLM. First, it is more sensitive to the temporal order of observations, as it takes history into account. For example, it treats differently two consecutive inconsistencies as the first one is more surprising than the second one, while the standard GLM treats them in the same way. This makes our approach more sensitive, more powerful (statistically) and more relevant to our research question. The second advantage is that we can estimate the hidden variables of AC/similarity which the standard GLM cannot. This allowed our model to identify the dMPFC area, which is involved in the tracking of AC.

### Limitations

One key limitation of the current study is that our task did not allow us to collect trial-by-trial behavioural data showing what participants had learnt about the agents. This is because we wanted participants to learn implicitly, rather than making explicit predictions of the agent’s choice on each trial. Because of this, we approximated a learning rate (0.5) and used it in our RL models to track changes in preference tracking according to the actual choices made by the agents. This raises the possibility that there may only be a weak fit between the learning rate used in our model and the actual learning rate of our participants. However, our main predictions related to the direction of the tracked PEs and accumulated preferences, and not with the specific magnitude of these variables, are less likely to be affected by our approximation. This is in line with a recent theoretical paper (Wilson and Niv, 2015) that demonstrated that model-based fMRI results are, under some conditions, insensitive to changes in individual learning rates. While it is possible that our approximation may lead to lower power at detecting brain responses to PEs, we feel that the main hypothesis concerning the direction of the effects (similarity approach *vs* consistency approach) is supported by our analysis.

## Conclusions

In this study, we combined computational modelling and fMRI to investigate the neural processes that underlie learning about the similarity of other people’s preferences to one’s own. We found that more regions of the brain encode information about the similarity of others’ choices in a consistency driven manner than encode that information purely based on each particular preference’s similarity to one’s own. This was particularly the case for the accumulated information about the other’s similarity with no areas showing sensitivity to purely accumulated similarity while a region of the dmPFC showed significant tracking of AC.

These findings suggest that higher level neural representations of similarity to the self are coded in a person-specific manner, which reflects how consistent are that person’s preference related to the self, i.e. do we usually agree or disagree in our preferences. As such our study highlights the role of context-dependent predictive processing in the learning of preference similarity between self and others and, by extension, in the formation of social impressions more generally. Further research in this area could build on our results by examining whether the neural correlates of similarity learning are modulated by having pre-existing cues about how similar that person is to oneself. In addition, it is possible that this consistency approach also applies to learning about other domains including people’s traits, attitudes and competence.

## Supplementary Material

scan-19-061-File007_nsz076
