# Supplementary material for: The neural basis of shared preference learning
Source: Soc Cogn Affect Neurosci. 2019 Nov 4;14(10):1061–72. doi: 10.1093/scan/nsz076 (PMC6970152; doi:10.1093/scan/nsz076)
Supplement: scan-19-061-File007_nsz076 [file scan-19-061-file007_nsz076.docx]

## The Neural Basis of Shared Preference Learning – Supplementary Materials

## S1. Supplementary Methods

### S1.1. Materials

The picture stimuli used in this study comprised of 40 pairs of abstract paintings and 40 pairs of landscape paintings that were matched as closely as possible in terms of their visual and aesthetic properties. This was done to ensure that different pairs of paintings would not differ wildly in their characteristics so that the choices of the different agent would not suggest an abnormal set of preferences. To construct these sets 120 abstract and 120 landscape images were downloaded from the internet and resized to 390x390 JPEG images with any remaining space on either dimension filled in with black. These images were then rated in a pre-study by a group of 20 participants on their complexity, concreteness, attractiveness, valence, affectivity and interest using a 7-point scale. In addition each images’ luminance and contrast were calculated using MATLAB (Mathworks 2015).

The mean ratings and luminance and contrast measures for each image were standardised across all images. Similarity scores were created for each measure by subtracting each images score from the score of every other image of its group (abstract or landscape). The similarity scores for all measures were then combined into one value using the following algorithm:

$$\left( Attractiveness*2 \right)+ \left( Interest*2 \right)+Complexity+Valance+Affectivity+\left( \frac{Concreteness}{2} \right)+\left( \frac{Luminance}{2} \right)+\left( \frac{Contast}{2} \right)$$

Each of the images was then paired with its closest neighbour and each pair was then removed from the array. The 40 closest pairs in each group were used in the fMRI experiment and the next 5 closest pairs were used in the training block.

### S1.2 Pre Scanning Training

Prior to entering the scanner participants completed a training block of the task consisting of 10 trials, to ensure that they understood the task. In the training block, they saw two agents of the opposite gender to themselves, and choices were made in the order: Agent 1, Participant, Agent 2. This was to create the belief that the order of making choices was random and that each agent made choices independently. In fact, in the experimental trials, the choices of the agents were determined from the participant choices to create appropriate levels of similarity. After the training, participants learnt the names of the agents with whom they would do the experimental task and rated their faces for similarity, likeability and attractiveness, using a 10-point scale. In the experimental blocks, participants saw these two agents of the same gender as them and the participants’ choice always came before the choices of the two agents. Images of the agents were taken from the Karolinska Directed Emotional Face database (Lundqvist, Flykt, & Öhman, 1998) but participants were informed that these photos were stand in images for the actual other participants.

### S1.3 Image Acquisition and Data Analysis

A 1.5 T Siemens TIM Avanto scanner with a 32-channel head coil was used to acquire both T1-weighted structural images and T2*-weighted echoplanar images using the multiband method (64×64 pixels; 3.2×3.2 mm; echo time, 55 ms, multiband factor=2) with blood oxygen level-dependent (BOLD) contrast. Each volume comprised 40 axial slices (3.2 mm thick, oriented approximately to the anterior commissure–posterior commissure plane), covering most of the brain but omitting inferior portions of the cerebellum. Functional scans were acquired in four sessions, each comprising 222 volumes (~7.4 min). Volumes were acquired continuously with an effective repetition time of 2s per volume. The first four volumes in each session were discarded to allow for T1 equilibration effects. Prior to functional scanning, a 6 min T1-weighted MPRAGE structural scan was collected at a resolution of 1×1×1 mm. Stimuli were projected onto a screen behind the participant and viewed in a mirror. Participants responded using a 4-button response box. All stimuli were presented with Cogent running under Matlab2014, permitting synchronisation with the scanner and accurate timing of stimuli presentation.

Data were processed and analysed using SPM12 ([www.fil.ion.ucl.ac.uk/spm](http://www.fil.ion.ucl.ac.uk/spm)). The EPI images from all four sessions of each participant were realigned to a mean EPI image for that participant. Images in which the participant moved more than 1.5mm or had rotation of more than 1 degree were visually examined and if seen to contain artefacts were removed from the analysis and replaced with volumes interpolated from the preceding and subsequent images. No participant had artefacts in more than 5% of images. Each participant’s structural image was processed using a unified segmentation procedure combining segmentation, bias correction, and spatial normalization to the MNI template (Ashburner & Friston, 2005). The same normalization parameters were then used to normalize the EPI images. Finally, the images were spatially smoothed to conform to the assumptions of the GLM implemented in SPM12 by applying a Gaussian kernel of 8 mm FWHM.

For each of our two GLMs SPM12 was used to compute first level parameter estimates (beta) and t-contrast images (containing weighted parameter estimates) for each contrast at each voxel. To examine regions showing a main effect of agent similarity, two contrasts were carried out between the *outcome screen* regressors (ASim > ADiff, ADiff > ASim). In addition, to examine regions that tracked the RL model parameters in each model, conjunction images were calculated for each RL parameter (AS _ASim_ /AC_ASim_ ∩ AS _ADiff_/AC_ADiff_) and (PE_Sim _ASim_ /PE_Con _ASim_ ∩ PE_Sim _ADiff_/PE_Con _ADiff_).

For the group-level analysis, the first level images from all participants were subjected to two one-sample t-tests, one in the positive direction and the other in the negative direction. Images derived from these second level analyses were thresholded at p < 0.001, uncorrected. For each analysis, a separate Monte Carlo simulation implemented in 3dClustSim (Forman et al., 1995) was used to determine the correct cluster extent threshold needed for a whole brain cluster-wise significance level of p < 0.05. Anatomical Regions were determined using the AICHA atlas (Joliot et al., 2015) to for gray matter and the Tractography based Atlas of human brain connections Projection Network (Natbrainlab, Neuroanatomy and Tractography Laboratory) (Catani & de Schotten, 2012; de Schotten et al., 2011) for the white matter.

## S2. Alternative Non Parametric Data Analysis

In addition to our two parametric GLMs we conducted an additional analysis to examine to what extent the use of our parametric modulators shed additional light on our findings when compared with a more traditional factorial GLM.

### S2.1 GLM Design and Data Analysis

This GLM modelled BOLD activation during agent *outcome* screens categorised across the factors of agent (ASim, ADiff) and choice (agree (Ag), disagree (Dis)). Regressors of no interest modelled activity during the *self-choice* *outcome* screen, the *feedback* phase, the ratings periods, trials where participants failed to make a choice and the residual effects of head motion.

SPM12 was used to compute first level parameter estimates (beta) and t-contrast images (containing weighted parameter estimates) for each contrast at each voxel. To examine regions showing a main effect of agent similarity two t-contrasts were carried out between the *outcome screen* regressors (ASimAg + ASimDis > ADiffAg + ADiffDis , ADiffAg + ADiffDis > ASimAg + ASimDis). To examine regions showing a main effect of choice similarity two t-contrasts were carried out between the *outcome screen* regressors (ASimAg + ADiffAg > ASimDis + ADiffDis, ASimDis + ADiffDis > ASimAg + ADiffAg). Finally, to examine regions showing a main effect of choice consistency two t-contrasts were carried out between the *outcome screen* regressors (ASimAg + ADiffDis > ASimDis + ADiffAg, ASimDis + ADiffAg > ASimAg + ADiffDis).

For the group-level analysis, the first level images from all participants were subjected to one-sample t-tests. Images derived from these second level analyses were thresholded at p < 0.001, uncorrected. For each analysis, a separate Monte Carlo simulation implemented in 3dClustSim (Forman et al., 1995) was used to determine the correct cluster extent threshold needed for a whole brain cluster-wise significance level of p < 0.05. Anatomical Regions were determined using the AICHA atlas (Joliot et al., 2015) for gray matter and the Tractography based Atlas of human brain connections Projection Network (Natbrainlab, Neuroanatomy and Tractography Laboratory) (Catani & de Schotten, 2012; de Schotten et al., 2011) for white matter.

### S2.2 Factorial GLM Results

Full results of this GLM analysis can be seen below in table S1. As can be seen the results the activations for the choice main effects and the consistency are largely in with those of our parametric modulator GLMs. The Disagree > Agree contrast results showed activations equivalent to the clusters shown for areas that negatively tracked similarity prediction errors, the results for the Consistent > Inconsistent contrast showed significant activations for two of the three clusters we identified that positively tracked consistency PE and the results for the Inconsistent > Consistent contrast showed results largely consistent with areas negatively tracking consistency PE.

**Table S1.** Peak voxel coordinates in MNI space, z-values and cluster sizes for analyses of the outcome period in the Consistency GLM showing significant effects after cluster correction for conjunction analyses of the AS and PE parametric modulators. Same shading indicates local maxima in distinct anatomical regions within the same cluster, BA indicates Brodman Area, k indicates the cluster size threshold for whole brain significance of *p* < 0.05.

| **Region** | **Hem.** | **X** | **Y** | **Z** | **Z-Score** | **Cluster Size** |
| --- | --- | --- | --- | --- | --- | --- |
| **Disagree > Agree (k = 35)** |  |  |  |  |  |  |
| Lateral Occipital Gyrus (18) | L | -30 | -92 | 22 | 4.29 | 395 |
| Cuneus (18) | L | -12 | -88 | 16 | 3.30 |  |
| Lateral Occipital Gyrus (37) | R | 32 | -54 | -16 | 3.94 | 129 |
| Fusiform Gyrus (19) | R | 30 | -64 | -14 | 3.37 |  |
| Fusiform Gyrus (37) | R | 28 | -46 | -14 | 3.18 |  |
| Lingual Gyrus (17) | L | -6 | -78 | 8 | 3.85 | 233 |
| Lingual Gyrus (18) | L | -8 | -70 | -2 | 3.34 |  |
| Occipital Superior Gyrus (18) | R | 24 | -92 | 16 | 3.80 | 337 |
| Middle Occipital Gyrus (19) | R | 36 | -80 | 22 | 3.46 |  |
| Lateral Occipital Gyrus (19) | R | 28 | -82 | -16 | 3.78 | 70 |
| Lateral Occipital Gyrus (37) | L | -28 | -60 | -16 | 3.64 | 126 |
| Fusiform Gyrus (37) | L | -26 | -48 | -14 | 3.46 |  |
| **Consistent > Inconsistent (35)** |  |  |  |  |  |  |
| Corpus Callosum | L | -2 | 14 | 10 | 3.70 | 37 |
| Corpus Callosum | R | 16 | -6 | 28 | 3.69 | 35 |
| **Inconsistent > Consistent (35)** |  |  |  |  |  |  |
| Superior Temporal Sulcus (37) | R | 62 | -58 | 12 | 4.79 | 108 |
| Supramarginal Gyrus (40) | R | 58 | -42 | 46 | 3.92 | 192 |
| Interparietal Sulcus (7) | R | 28 | -50 | 42 | 3.82 | 65 |
| Superior Frontal Sulcus (10) | R | 34 | 50 | 10 | 3.61 | 62 |
| Precuneus | R | 8 | -58 | 48 | 3.56 | 99 |
| Middle Temporal Gyrus (20) | R | 62 | -24 | -14 | 3.49 | 61 |
| Middle Temporal Gyrus (21) | R | 64 | -18 | -8 | 3.28 |  |

However, there were two important differences which point to the increased value of our modelling analysis. First, the simple contrast GLM did not identify any areas that could be explained by agent identity alone as opposed to choice similarity, presumably because the close relationship between these two factors meant that the variance in activation was captured by the choice analysis instead. Second, this analysis does not capture our finding of the dMPFC area which is involved in the tracking of accumulated consistency. Thus while the prediction error aspects of our model based analysis can be captured by a more traditional analysis, our use of computational modelling has have additional value in giving greater insight into the mechanism behind such activations and in allowing us to additionally understand which brain areas are involved in the representation of the current predictions regarding the others consistency in similarity.

## S3. dmPFC literature comparison

To place our findings of accumulated consistency being tracked within the dmPFC in context, we identified 15 studies that had found activation in the dMPFC and categorised the contrasts those activations came from into the following four categories: 1) Diagnostic > Non-Diagnostic cases: contrasting information relevant to a trait judgement about an individual with irrelevant information; 2) Inconsistent > Consistent: cases contrasting novel information that was inconsistent with previous knowledge about an individual with novel information that was consistent with previous knowledge; 3) Other Impression Formation: other contrasts relevant to impression formation, often linking photographs of individuals with information about their traits; 4) Self Relevant: contrasts in which participants judged whether information was self-relevant or not (see Table S2). We then collapsed the clusters found in these studies across the x-axis to create Figure S1. Our result falls in the middle of the region activated by previous studies, with most contrasts investigating Inconsistent > Consistent falling more dorsally and most contrasts investigating Diagnostic > Non-Diagnostic falling more ventrally suggesting that the activation found in our study is in agreement with the previous literature.


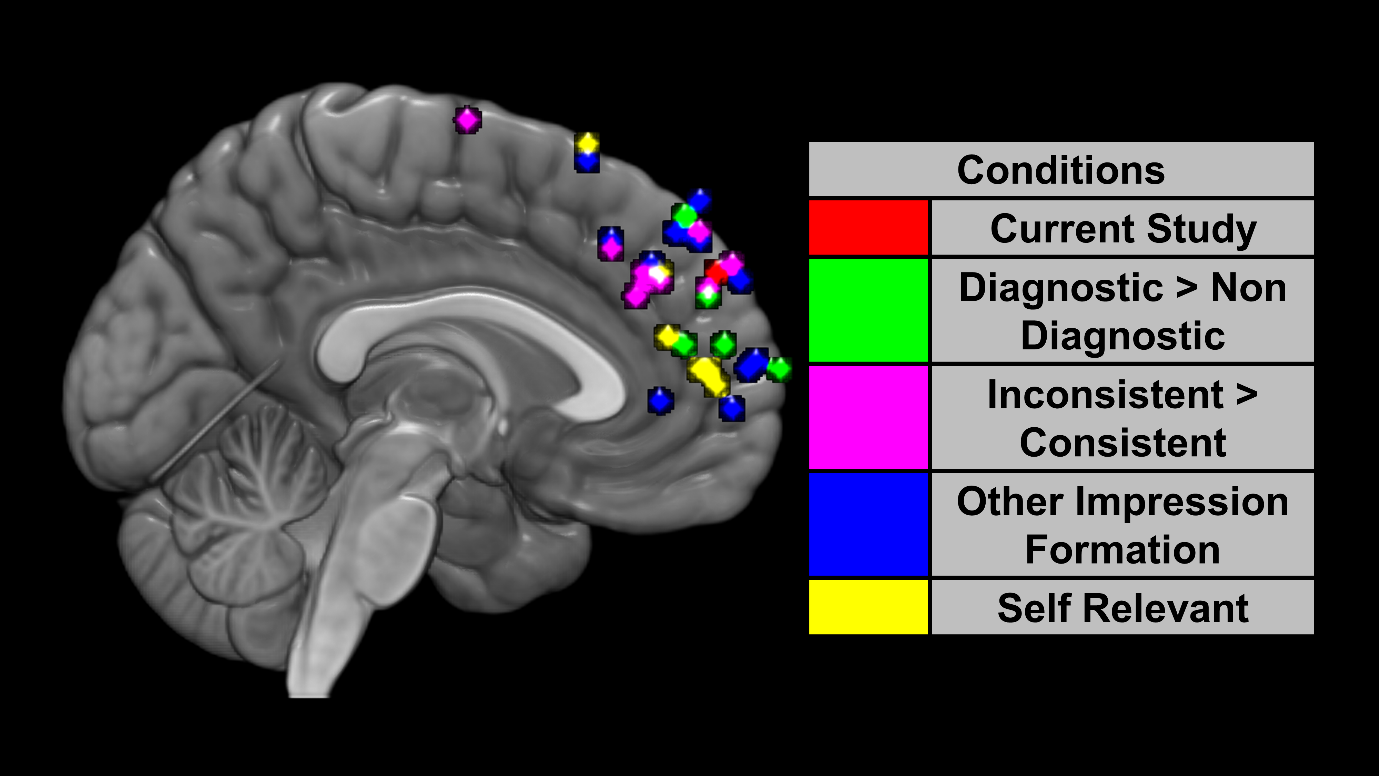
Figure S1. dmPFC activations across 15 studies investigating impression formation and the self together with the result from the current study. For ease of presentation we have collapsed the studies across the x-axis (max x = 14, min x = -13).

**Table S2.** Details of studies used in the comparison of dmPFC activation across impression formation studies (see Figure S1). Coordinates are reported in MNI space and coordinates from studies using Talairach space were converted using the WFU PickAtlas version 2.4 (Maldjian, Laurienti, and Burdette 2004; Maldjian et al. 2003).

| **Study** | **Information presented** | **Impression studied** | **X** | **Y** | **Z** |
| --- | --- | --- | --- | --- | --- |
| **Current Study (Red)** | Choice of preferred painting | Similarity to Self | 8 | 5 | 34 |
| **Diagnostic > Non Diagnostic (Green)** | |  |  |  |  |
| Gilron & Gutchess, 2012 | Moral and Neutral Behaviours | Moral Impression vs Location of Behaviour | 0 | 57 | 15 |
|  | Moral and Neutral Behaviours | Moral Impression vs Location of Behaviour | 3 | 48 | 48 |
|  | Moral and Neutral Behaviours | Moral Impression vs Location of Behaviour | 9 | 72 | 9 |
| Ma, Vandekerckhove, Van Overwalle, Seurinck, & Fias, 2010 | Moral and Neutral Behaviours | Personality Traits | 4 | 54 | 28 |
|  | Moral and Neutral Behaviours | Personality Traits | 14 | 48 | 16 |
| **Inconsistent > Consistent (Pink)** | |  |  |  |  |
| Cloutier, Gabrieli, Young, & Ambady, 2011 | Political Views | Political Affiliation | 2 | 54 | 30 |
|  | Political Views | Political Affiliation | 6 | 52 | 44 |
| Ma et al., 2012 | Moral vs Neutral Behaviours | Moral Traits | 4 | 42 | 32 |
|  | Moral vs Neutral Behaviours | Moral Traits | 4 | 38 | 34 |
|  | Moral vs Neutral Behaviours | Moral Traits | 4 | 35 | 28 |
| Mende-Siedlecki, Cai, & Todorov, 2013 | Moral Behaviours | Moral Traits | 2 | 31 | 40 |
| Mende-Siedlecki & Todorov, 2016 | Moral and Neutral Behaviours | Trustworthiness and Surprise | -5 | 59 | 36 |
|  | Moral and Neutral Behaviours | Trustworthiness and Surprise | -2 | -5 | 72 |
| **Other Impression Formation (Blue)** | |  |  |  |  |
| Ames & Fiske, 2013 | Assessment of Teaching Ability | Expertise | -4 | 45 | 45 |
| Baron, Gobbini, Engell, & Todorov, 2011 | Moral Behaviours | Trustworthiness | -4 | 41 | 36 |
| Freeman, Schiller, Rule, & Ambady, 2010 | Individuated vs Superficial for Racial In-group vs Out-group | Personality Traits | -13 | 43 | 4 |
| Fouragnan et al., 2013 | Choices in Trust Games and Prior information about Trustworthiness | Trustworthiness | -2 | 64 | 10 |
|  | Choices in Trust Games and Prior information about Trustworthiness | Trustworthiness | 0 | 62 | 31 |
| Gilron & Gutchess, 2012 | Moral Behaviours vs Neutral Behaviours | Moral Impession vs Location of Behaviour | 3 | 30 | 42 |
| Mende-Siedlecki, Baron, & Todorov, 2013 | Moral and Ability Behaviours | Competence and Trusworthiness | -5 | 66 | 13 |
|  | Moral and Ability Behaviours | Competence and Trusworthiness | 32 | 60 | -1 |
| Mende-Siedlecki, Cai, et al., 2013 | Moral Behaviours | Moral Impression | 5 | 52 | 51 |
| Schiller, Freeman, Mitchell, Uleman, & Phelps, 2009 | Moral Behaviours | Moral Impression | -9 | 24 | 61 |
| Schiller et al., 2009 | Moral Behaviours | Moral Impression | -7 | 52 | 43 |
| **Self (Yellow)** | |  |  |  |  |
| Martinelli et al. 2013 | Memory Meta-Analysis | Semantic Autobiographic Memory | -10 | 45 | 18 |
|  | Memory Meta-Analysis | Episodic Autobiographic Memory | -6 | 51 | 9 |
|  | Memory Meta-Analysis | Conceptual Self | 6 | 55 | 5 |
| Moran, J.M. et al., 2006 | Personality Traits | Self-Relevance | -6 | 53 | 6 |
| Phan, K.L. et al., 2004 | Emotional Pictures | Self-Relatedness | 0 | 42 | 33 |
| Schneider et al. 2008 | Emotional or Neutral Pictures | Self-Relatedness | -3 | 24 | 66 |

## Supplementary References

Ashburner, J., & Friston, K. J. (2005). Unified segmentation. *NeuroImage*, *26*(3), 839–851.

Ames, Daniel L, and Susan T Fiske. 2013. “Outcome Dependency Alters the Neural Substrates of Impression Formation.” *NeuroImage* 83: 599–608.

Baron, Sean G, Maria Ida Gobbini, Andrew D. Engell, and Alexander Todorov. 2011. “Amygdala and Dorsomedial Prefrontal Cortex Responses to Appearance-Based and Behavior-Based Person Impressions.” *Social Cognitive and Affective Neuroscience* 6 (5): 572–81.

Brett, Matthew, Jean-Luc L Anton, Romain Valabregue, and Jean-Baptiste Poline. 2002. “Region of Interest Analysis Using an SPM Toolbox.” *NeuroImage* 16 (2.1): 1140.

Catani, M., & de Schotten, M. T. (2012). *Atlas of human brain connections*. Oxford: Oxford University Press.

Cloutier, Jasmin, J D E Gabrieli, D O Young, and Nalini Ambady. 2011. “An fMRI Study of Violations of Social Expectations: When People Are Not Who We Expect Them to Be.” *NeuroImage* 57 (2): 583–88.

de Schotten, M. T., Ffytche, D. H., Bizzi, A., Dell’Acqua, F., Allin, M., Walshe, M., … Catani, M. (2011). Atlasing location, asymmetry and inter-subject variability of white matter tracts in the human brain with MR diffusion tractography. *NeuroImage*, *54*(1), 49–59.

Forman, S. D., Cohen, J. D., Fitzgerald, M., Eddy, W. F., Mintun, M. A., & Noll, D. C. (1995). Improved assessment of significant activation in functional magnetic resonance imaging (fMRI): Use of a cluster-size threshold. *Magnetic Resonance in Medicine*, *33*(5), 636–647.

Fouragnan, Elsa, Gabriele Chierchia, Susanne Greiner, Remi Neveu, Paolo Avesani, and Giorgio Coricelli. 2013. “Reputational Priors Magnify Striatal Responses to Violations of Trust.” *Journal of Neuroscience* 33 (8): 3602–11.

Freeman, Jonathan B, Daniela Schiller, Nicholas O Rule, and Nalini Ambady. 2010. “The Neural Origins of Superficial and Individuated Judgments about Ingroup and Outgroup Members.” *Human Brain Mapping* 31 (1): 150–59.

Gilron, Roee, and Angela H Gutchess. 2012. “Remembering First Impressions: Effects of Intentionality and Diagnosticity on Subsequent Memory.” *Cognitive, Affective and Behavioral Neuroscience* 12 (1): 85–98.

Joliot, M., Jobard, G., Naveau, M., Delcroix, N., Petit, L., Zago, L., … Tzourio-Mazoyer, N. (2015). AICHA: An atlas of intrinsic connectivity of homotopic areas. *Journal of Neuroscience Methods*, *254*, 46–59.

Lundqvist, D., Flykt, A., & Öhman, A. (1998). The Karolinska directed emotional faces. Stockholm: Department of Clinical Neuroscience, Psychology section, Karolinska Institutet.

Ma, Ning, Marie Vandekerckhove, Kris Baetens, Frank Van Overwalle, Ruth Seurinck, and Wim Fias. 2012. “Inconsistencies in Spontaneous and Intentional Trait Inferences.” *Social Cognitive and Affective Neuroscience* 7 (8): 937–50.

Ma, Ning, Marie Vandekerckhove, Frank Van Overwalle, Ruth Seurinck, and Wim Fias. 2010. “Spontaneous and Intentional Trait Inferences Recruit a Common Mentalizing Network to a Different Degree: Spontaneous Inferences Activate Only Its Core Areas.” *Social Neuroscience* 6 (2): 123–38.

Maldjian, Joseph A, Paul J Laurienti, and Jonathan H Burdette. 2004. “Precentral Gyrus Discrepancy in Electronic Versions of the Talairach Atlas.” *NeuroImage* 21 (1): 450–55.

Maldjian, Joseph A, Paul J Laurienti, Robert A Kraft, and Jonathan H Burdette. 2003. “An Automated Method for Neuroanatomic and Cytoarchitectonic Atlas-Based Interrogation of fMRI Data Sets.” *NeuroImage* 19 (3): 1233–39.

Martinelli, Pénélope, Marco Sperduti, and Pascale Piolino. 2013. “Neural Substrates of the Self-Memory System: New Insights from a Meta-Analysis.” *Human Brain Mapping* 34 (7): 1515–29.

Mathworks. 2015. “Matlab R2015b.” Natick, MA: The Mathworks Inc.

Mende-Siedlecki, Peter, Sean G Baron, and Alexander Todorov. 2013. “Diagnostic Value Underlies Asymmetric Updating of Impressions in the Morality and Ability Domains.” *Journal of Neuroscience* 33 (50): 19406–15.

Mende-Siedlecki, Peter, Yang Cai, and Alexander Todorov. 2013. “The Neural Dynamics of Updating Person Impressions.” *Social Cognitive and Affective Neuroscience* 8 (6): 623–31.

Mende-Siedlecki, Peter, and Alexander Todorov. 2016. “Neural Dissociations between Meaningful and Mere Inconsistency in Impression Updating.” *Social Cognitive and Affective Neuroscience* 11 (9): 1489–1500.

Moran, Joseph M, C Neil Macrae, Todd F Heatherton, C L Wyland, and W M Kelley. 2006. “Neuroanatomical Evidence for Distinct Cognitive and Affective Components of Self.” *Journal of Cognitive Neuroscience* 18 (9): 1586–94.

Phan, K Luan, Stephan F. Taylor, Robert C. Welsh, Shao Hsuan Ho, Jennifer C. Britton, and Israel Liberzon. 2004. “Neural Correlates of Individual Ratings of Emotional Salience: A Trial-Related fMRI Study.” *NeuroImage* 21 (2): 768–80.

Schiller, Daniela, Jonathan B Freeman, Jason P Mitchell, James S Uleman, and Elizabeth A Phelps. 2009. “A Neural Mechanism of First Impressions.” *Nature Neuroscience* 12 (4): 508–14.

Schneider, Felix, F. Bermpohl, A. Heinzel, M. Rotte, M. Walter, C. Tempelmann, C. Wiebking, H. Dobrowolny, H. J. Heinze, and G. Northoff. 2008. “The Resting Brain and Our Self: Self-Relatedness Modulates Resting State Neural Activity in Cortical Midline Structures.” *Neuroscience* 157 (1): 120–31.
